# Exploring Reasons for Choosing Psychiatry Among Psychiatrists in the United Arab Emirates

**DOI:** 10.1192/bjo.2022.131

**Published:** 2022-06-20

**Authors:** Syed Fahad Javaid, Leena Amiri, Fadwa Al Mugaddam, Hind Mohd Ahmed, Amani Alkharoossi

**Affiliations:** 1 Department of Psychiatry and Behavioural Sciences, College of Medicine and Health Sciences, United Arab Emirates University, Al Ain, UAE.; 2 Behavioural Sciences Institute, Al Ain Hospital, Al Ain, UAE

## Abstract

**Aims:**

The global burden of mental disorders continues to grow with significant health, social and economic consequences. Unfortunately, the gap between the need for mental health care and its provision remains wide all over the world. The recruitment and retention of psychiatrists is a long-standing concern in the United Arab Emirates (UAE), with social stigma playing a potential role. This study aimed to investigate the factors that affect the choice of psychiatry as an area of practice by psychiatrists in the UAE. A secondary aim was to assess differences in the factors which affected career decisions among those participants with different backgrounds to establish any cultural and generational differences in choosing psychiatry as a career.

**Methods:**

We conducted correlational research using an anonymised 30-item online questionnaire. We recruited qualified psychiatrists currently working in the UAE. The structured questionnaire assessed the participants’ sociodemographic factors and reasons for choosing psychiatry. Ethical approval was received from the Social Sciences Research Ethics Committee (SS-REC) at United Arab Emirates University. Statistical analysis, including Pearson correlations and chi-square tests, was performed using the statistical package for the social sciences (SPSS) version 26.

**Results:**

Out of 70 participants approached, 54 completed the questionnaire with a response rate of 77%. 69% of the participants were female, with a mean age of 38 years. 46% were UAE citizens. We found that the doctors trained in the UAE were statistically more likely to face opposition to specialising in psychiatry (p-value <0.001). Participants with a family member or friend as a psychiatrist were more likely to choose psychiatry as a first choice (p-value 0.01). Psychiatrists below the age of 35 were more statistically likely to face opposition to their decision to specialise in psychiatry (p-value 0.006). Psychiatrists who regretted their decision to specialise in psychiatry were statistically more likely to feel this way in their first year of residency (p-value <0.001). This study had its limitations, including the generalisability of findings and the sociodemographic factors of participants. The self-reported methodology could have subjected findings to bias, including social desirability bias.

**Conclusion:**

Multiple sociodemographic factors influence the decision to specialise in psychiatry in the UAE. These findings would be helpful to identify hurdles faced by the young UAE clinicians in choosing psychiatry, partly explaining the dearth of UAE-trained psychiatrists in the country. Further research is required to study these reasons in detail, helping to improve the recruitment and retention of UAE psychiatrists in the future.

